# Airborne SARS-CoV-2 is more frequently detected in environments related to children and elderly but likely non-infectious, Norway, 2022

**DOI:** 10.1186/s12985-023-02243-4

**Published:** 2023-11-24

**Authors:** Priscilla Gomes da Silva, Mahima Hemnani, José Gonçalves, Elisa Rodriguéz, Pedro A. García-Encina, Maria São José Nascimento, Sofia I. V. Sousa, Mette Myrmel, João R. Mesquita

**Affiliations:** 1 ICBAS-School of Medicine and Biomedical Sciences, Porto University, Porto, Portugal; 2 Epidemiology Research Unit (EPIunit), Institute of Public Health, University of Porto, Porto, Portugal; 3 Laboratório para a Investigação Integrativa e Translacional em Saúde Populacional (ITR), Porto, Portugal; 4 LEPABE-Laboratory for Process Engineering, Environment, Biotechnology and Energy, Faculty of Engineering, University of Porto, Porto, Portugal; 5 ALiCE-Associate Laboratory in Chemical Engineering, Faculty of Engineering, University of Porto, Porto, Portugal; 6 Institute of Sustainable Processes, Valladolid University, Dr. Mergelina s/n, Valladolid, 47011 Spain; 7 Faculty of Pharmacy, University of Porto, Porto, Portugal; 8 Virology Unit, Norwegian University of Life Sciences, Ås, Norway

**Keywords:** SARS-CoV-2, Airborne transmission, COVID-19, Indoor environments, Outdoor environments, Mitigation strategies, Vaccination, Mask mandates

## Abstract

This study investigates the presence of SARS-CoV-2 in indoor and outdoor environments in two cities in Norway between April and May 2022. With the lifting of COVID-19 restrictions in the country and a focus on vaccination, this research aims to shed light on the potential for virus transmission in various settings. Air sampling was conducted in healthcare and non-healthcare facilities, covering locations frequented by individuals across different age groups. The study found that out of 31 air samples, only four showed the presence of SARS-CoV-2 RNA by RT-qPCR, with no viable virus detected after RNAse pre-treatment. These positive samples were primarily associated with environments involving children and the elderly. Notably, sequencing revealed mutations associated with increased infectivity in one of the samples. The results highlight the importance of considering children as potential sources of virus transmission, especially in settings with prolonged indoor exposure. As vaccination coverage increases globally, and with children still representing a substantial unvaccinated population, the study emphasizes the need to re-implement mask-wearing mandates indoors and in public transport to reduce virus transmission. The findings have implications for public health strategies to control COVID-19, particularly in the face of new variants and the potential for increased transmission during the autumn and winter seasons.

## Introduction

Almost three years have passed since the COVID-19 pandemic started in December 2019 [1]. During this time, much has been debated about the transmission of SARS-CoV-2, and how each transmission mode contributed to the worldwide spread of the virus, as well as all the factors involved in different transmission settings [2–4]. Now, it is well established that transmission through surfaces did not account for a great proportion of the infections, and that transmission occurs mainly through close contact, respiratory droplets and aerosols [5, 6], with indoor airborne transmission likely being the main driver of the pandemic [7–10].

Strict vaccination, surveillance and control policies helped to control viral spread, however, there is still a major gap in knowledge when it comes to airborne transmission, namely: how different environmental and meteorological parameters affect virus infectivity in the environment; how long the virus survives in the air; and more importantly, what is the minimum infectious dose of the virus. This information is essential for understanding airborne transmission dynamics and how we can prevent infection more efficiently [10–12]. There are several inherent difficulties in studying airborne SARS-CoV-2, e.g. the low virus concentration in air compared to clinical samples from the nasopharynx [13]; the variation of air-sampling techniques used, making generalization and interpretation of results difficult [14]; the lack of BSL-3 facilities for assessment of viral viability in air in most of the studies [15, 16], among others.

Although a high vaccination coverage was reached in most of Europe and America by the first trimester of 2022 [17], still new waves with record-high number of cases have been reported in that year, with the surge of a more transmissible variant of concern named Omicron (B.1.1.529) and its sub-lineages BA.4 and BA.5 31 [18]. Since distinct vaccine booster strategies are in place across the world that cover different age groups and individuals with different risk profiles, information regarding viral presence in air can provide a more in-depth characterization of the risk for COVID-19 infection.

In Norway, in early 2022, 86% of individuals above 16 years of age were vaccinated with two doses, and 86% of people ≥ 65 years old had received a booster dose [19]. No COVID-19 restrictions were in place other than the use of mask in healthcare facilities. As of 9th January 2022 in Norway, 72% of the entire population have been fully vaccinated. 86% of the people in the age group 16 years-old or older, and 88% of the people in the age group 18 years-old or older were fully vaccinated. Moreover, 82% of the people in the 16–17 years-old age group and 52% of people in the 12–15 years-old age group had been vaccinated with one dose, and 35% of people in the 16–17 years-old age group had been fully vaccinated [19].

With that in mind, we have performed air sampling in two cities in Norway in April and May 2022 in order to assess SARS-CoV-2 presence in different indoor and outdoor environments. Healthcare and non-healthcare related facilities were included, covering locations frequented by people of all age groups. Considering the complete lift of all restrictions in the country during that period, and the fact that the country had a low number of new daily infections, these results can be used as indicator of potential virus spread rates and the level of transmission risk in the country during that period.

## Materials and methods

### Air sampling

Air sampling (n = 31) was performed in the period of two weeks, between April and May 2022, in indoor and outdoor areas of two cities of Norway, Ås and Oslo, in healthcare and non-healthcare facilities, at periods of the day with increased movement of people.

Air samples were collected using the Coriolis® Compact (Bertin Instruments, Montigny-le-Bretonneux, France) air sampler with an airflow rate of 50 L/min. The duration of sampling was set according to the type of environment and permissions obtained for sampling, with slightly shorter sampling duration indoors where a high number of people was present. The minimum distance between the air sampling device and potential emission sources was 0.5 m in all sampling locations. The maximum distance varied depending on the size of the location and whether it was an open space or not, with distances of over 3 m between the air sampler and potential emission sources in open spaces such as the public parks and squares. Details about air sampling locations and settings are summarized in Table 1.

**Table 1:** Details about the air sampling settings and sampling sites

	Date	Sample number	Environment	Sampling site	Duration of sampling (min)	Period of the day
Non-healthcare facilities	24 April	1	Outdoor	Oslo Central Station	30	7:00–8:30am
		2	Outdoor	Statue in front of Oslo Central Station	30	8:30−9:00am
		3	Outdoor	Public Park	30	14:00–14:30pm
		4	Outdoor	Public Park	30	15:30−16:00pm
		5	Outdoor	Raadhus square	30	17:00–17:30pm
	25 April	6	Outdoor	Oslo Central Station	30	7:00–7:30am
		7	Outdoor	Statue in front of Oslo Central Station	30	8:30−9:00am
		8	Outdoor	Tram station	30	9:30−10:00am
		9	Outdoor	Public Park	30	10:30−11:00am
		10	Outdoor	Parliament square	30	13:00–13:30pm
	26 April	11	Outdoor	Oslo Central Station	30	7:00–7:30am
		12	Indoor	University Restaurant	50	11:50−12:40am
		13	Indoor	University Cafeteria	50	13:00–13:50pm
	27 April	14	Outdoor	Oslo Central Station	30	7:00–7:30am
		15	Indoor	University Restaurant	50	11:50−12:40am
		16	Indoor	University Cafeteria	50	13:00–13:50pm
	28 April	17	Outdoor	Oslo Central Station	30	7:00–7:30am
		18	Indoor	University Restaurant	50	11:50−12:40am
		19	Indoor	University Cafeteria	50	13:00–13:50pm
	29 April	20	Outdoor	Oslo Central Station	30	7:00–7:30am
		21	Indoor	Kindergarten	20	10:00–10:20am
		22	Outdoor	Kindergarten	20	10:40am-11:00am
		23	Indoor	Kindergarten	20	11:20am-11:40am
Healthcare facilities	02 May	24	Indoor	Nursing home	20	9:00–9:20am
		25	Indoor	Nursing home	20	9:40−10:00am
		26	Indoor	Nursing home	20	10:20−10:40am
		27	Indoor	Nursing home	20	11:00 −11:20am
		28	Indoor	Health Clinic	20	16:00–16:20pm
		29	Indoor	Health Clinic	20	16:40−17:00pm
		30	Indoor	Health Clinic	20	17:20−17:40pm
		31	Indoor	Health Clinic	20	18:00–18:20pm

The sampler was placed at a height of approximately 1 m at all sampling sites. Air samples were collected on a dry medium, with 1.5 mL of sterile phosphate buffered saline (PBS) added to the collection cones after sampling. All samples were stored at 4 °C and transported to the laboratory within 4 h, where they were stored at – 80 °C until further processing.

### RNA extraction and RT-qPCR

RNA extraction was performed using the GRS Viral DNA/RNA Purification Kit (GRISP, Porto, Portugal) according to the manufacturer’s instructions using 200 µL of sample suspension, resulting in 50 µL of RNA eluate after extraction as previously described [20]. Two RT-qPCR reactions targeting N1 and N2 were used (Xpert qDetect COVID-19, GRISP, Porto, Portugal). The CFX Real-Time PCR (qPCR) Detection System (Bio-Rad, USA) with the Bio-Rad CFX Maestro 1.0 Software version 4.0.2325.0418 was used for data analysis. For each reaction, 4 µL of RNA was used, with every run including ssDNA N1 and N2 targets (positive controls) and a no-template control. Reactions were run for 15 min at 45 °C and 2 min at 95 °C, then 45 cycles of 95 °C for 15 s, and 55 °C for 30 s. All samples were run in duplicates.

A standard curve was generated using ssDNA targets for both N1 and N2 in a 10-fold serial dilution starting at 200,000 copies/µL, in order to quantify viral gene copies based on sample Ct values. The limit of detection (LOD) was 1.3 and 3.2 copies/µL for N1 and N2, respectively. Air sampling results are expressed in gene copies/m^3^.

### Viral viability

The viral viability was evaluated with a nuclease sample treatment prior to RNA extraction using a previously described method with minor modifications [21]. The RNAse pre-treatment assumes that viable viruses with intact capsids and envelopes will not have their genetic material degraded by RNAse and amplification during the RT-qPCR would indicate viable viruses [22]. Prior to RNA extraction, three aliquots of the air sample were pre-treated with 8 µL of RNAse A (GRISP, Porto, Portugal) with the following concentrations: 1 µg/µL, 10 µg/µL and 50 µg/µL, at 37 °C for 30 min. An untreated aliquot of each sample was also tested for comparison of RT-qPCR results between treated and non-treated samples.

### Sequencing

Positive RT-qPCR samples were confirmed by heminested RT-PCR targeting a 398-bp fragment of the SARS-CoV-2 N gene [23]. Amplicons of expected sizes in the gel were purified with GRS PCR Purification Kit (Grisp, Porto, Portugal) and, bidirectional Sanger sequencing was performed with the specific primers of the target gene. Sequences were aligned with BioEdit Sequence Alignment Editor v7.1.9 software package, version 2.1 (Ibis Biosciences, Carlsbad, CA, USA) and compared with the sequences available in the NCBI (GenBank, Carlsbad, CA, USA) nucleotide database (http://blast.ncbi.nlm.nih.gov/Blast, accessed on 9th November 2022).

## Results

Out of 31 samples, four were positive for SARS-CoV-2 RNA by RT-qPCR when pre-treatment with RNAse was not performed. These samples were: sample 21 (kindergarten, indoor eating room) [6 gene copies/m^3^], sample 22 (kindergarten, outdoor playground) [6 gene copies/m^3^], sample 27 (nursing home, indoor cafeteria) [6 gene copies/m^3^] and sample 28 (Health clinic, pediatrics waiting room) [5 gene copies/m^3^]. Pre-treatment with the three RNAse concentrations followed by RT-qPCR showed that all the previously positive samples were negative.

**Fig. 1:**
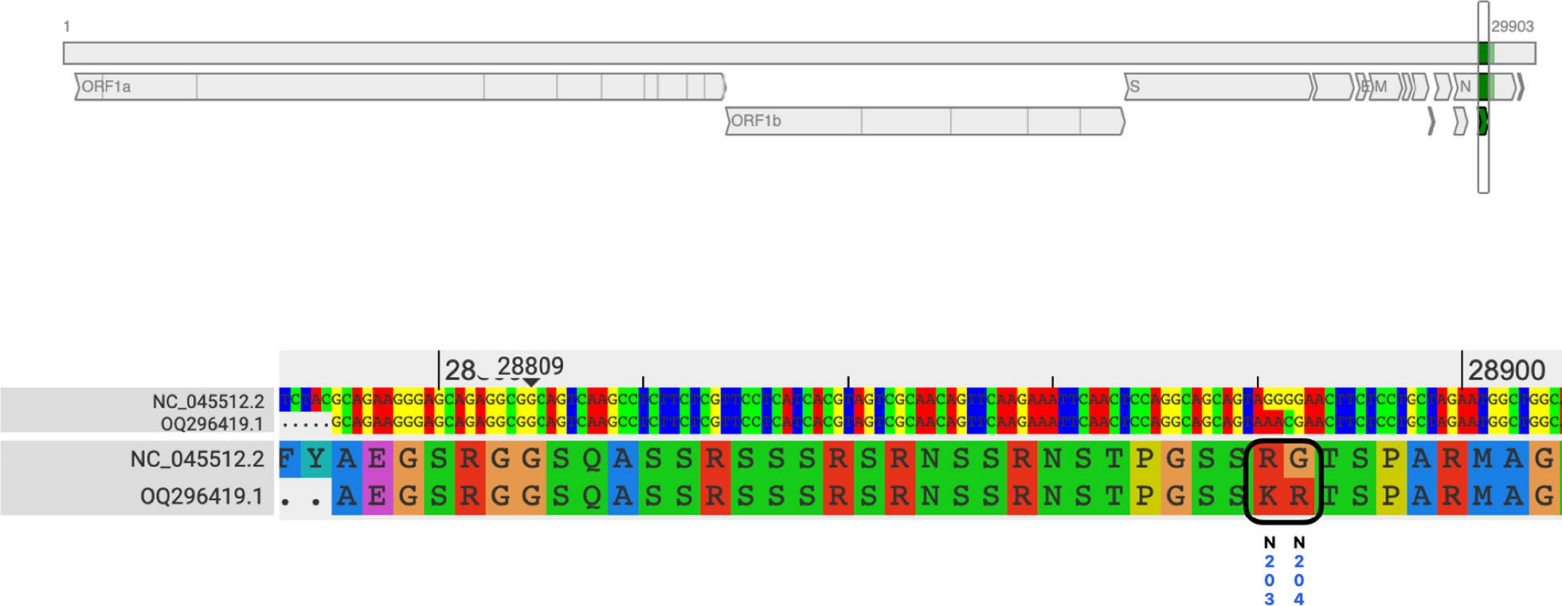
Alignment and genome annotations for the sequenced sample with the SARS-CoV-2 isolate Wuhan-Hu-1 (NC_045512.2) highlighting the R203K and G204R mutations. Modified from Coronavirus Typing Tool, Version 1.25

Heminested RT-PCR followed by bidirectional sequencing allowed to retrieve one sequence. This sequence was obtained from sample 21 (GenBank accession number OQ296419). BLAST analysis showed 100% identity with a SARS-CoV-2 isolate from a human from Tokyo, Japan (OQ326844). Genomic sequence analysis identified two mutations in relation to the SARS-CoV-2 strain first detected in Wuhan in (NC_045512.2), both in the nucleocapsid (R203K and G204R) (Fig. 1).

## Discussion

With all COVID-19 restrictions being lifted in Norway, and no obligation for mask use, not even in healthcare facilities, the present study aimed to assess the presence of SARS-CoV-2 in air samples from various indoor and outdoor spaces, including both healthcare and non-healthcare related facilities. We found that, out of 31 air samples, only four showed the presence of SARS-CoV-2 RNA by RT-qPCR, with no amplification after RNAse pre-treatment, strongly suggesting that no viable virus was present in these four samples.

The four positive samples came from a kindergarten (n = 2), a nursing home (n = 1) and from the pediatrics waiting room in a health clinic (n = 1). It should be noted that on the day of the sampling in the kindergarten, most of the children were showing respiratory disease symptoms but none was using facemasks. Moreover, at the nursing home one of the nurses’ staff had tested positive for COVID-19 the previous day.

It is important to mention that the number of people present in each sampled location varied throughout sampling duration in every location, as sampled locations were either outdoor public spaces with a constant and heavy influx of people such as public parks or the train station, or indoor spaces with constant but not so heavy movement of people such as the health clinic, where patients would come and go constantly. Nonetheless, in the locations where SARS-CoV-2 RNA could be recovered from the air samples, the density of people present was low (that is, less than 15 people present at the locations during sampling).

When looking at these results, although they indicate that non-infectious viruses were present in air samples, we cannot exclude the possibility that the collection process might have contributed to the inactivation of SARS-CoV-2 [24]. Multiple factors can potentially contribute to the decline in virus viability, including the collection process and prolonged airborne state (which increases the likelihood of surface damage due to impaction, exposure to harmful airborne contaminants, desiccation, or degradation) [25]. In the case of the Coriolis Compact, which is a cyclone sampler, a film of liquid is injected close to the cyclone’s inlet to wet the cyclone walls, which are subsequently collected at the cyclone’s base for analysis. This is thought to increase viability of collected viral or other microorganism particles that are being sampled, however shear forces may still decrease particles ‘viability [26–28].

As the probability of detecting infectious SARS-CoV-2 is directly correlated with the amount of viral RNA detected by RT-qPCR, and infectious viruses are more likely to be detected when viral RNA is present in concentrations greater than 1 × 10^6^ gene copies/mL [29], viable viruses were not expected to be present in our study based on the low numbers of gene copies/m^3^ found. This is in keeping with previous studies on SARS-CoV-2 that identified viable virus in air samples [30–32], with 1000 gene copies/m^3^ [31]. Of note, in another study it was estimated that an RNA concentration of 2.5 × 10^5^ RNA copies/mL had less than a 5% success rate for isolating infectious virus [33]. Within this < 5% success rate of isolating infectious virus, another study reported a limit of 4.3 × 10^6^ RNA copies/mL [34]. All these values are in accordance with other studies which have shown that samples with Ct values > 24 are unlikely to be virus positive after cultured [35]. However, it is important to emphasize that Ct measurements depend on the RT-qPCR assay and platform used [36].

Information about viability of SARS-CoV-2 viruses in air samples is very important when discussing risk assessment, as risk assessment studies are used by policymakers and health agencies to develop mitigation and preventative strategies to control viral transmission in the community, as well as its applications in occupational health [37]. In this context, the nuclease pre-treatment method offers an alternative to estimate the presence of infectious viruses in air samples when viral culture is not possible, allowing for a better interpretation of studies on SARS-CoV-2 presence in ar. Moreover, this method has the potential to enhance analyses aimed at supporting risk-based investigations, whether for preventing new outbreaks or managing recurrent ones, and can be applied across a wide range of scenarios [38].

SARS-CoV-2 typically causes mild illness and few deaths in children and adolescents when compared to adults [39]. However, these groups still remain susceptible to SARS-CoV-2 infection and may transmit the virus to other people, such as the elderly parcel of the population, which increases the burden of the disease on public health systems (World Health Organization (WHO) [40]). Interestingly, our results point to a link between SARS-CoV-2 presence in air in environments with more children under the age of three present, which, until this day, still makes up the age-group worldwide with more unvaccinated people against COVID-19 [41, 42].

Nonetheless, the sequenced sampled presented the nucleocapsid mutations R203K (28881G >A, 28882G>A) and G204R (28883G>C) that have been reported to increase the infectivity, fitness, and virulence of SARS-CoV-2 [43], being associated with increased infectivity of SARS-CoV-2 strains in the United States (USA), as well as being predominant in both the USA and Europe [44]. The mutation G204R is non-conservative, and the R203K mutation has been pointed to function as a non-conservative substitution due to the different size of the arginine (R) versus lysine (K) residues and the considerably different chemical features of the side-chain guanidinium group (arginine) versus the primary amine (lysine). It has been hypothesized that these mutations may influence disease severity by altering linker region flexibility and dynamics, which would in turn alter nucleocapsid function [45].

One of the significant unanswered questions about the COVID-19 epidemiology is related to the role of children in the transmission of SARS-CoV-2 [46], which is a group that comprises a significant share of the population in many countries (Charumilind et al. [47]). With milder symptoms, children are tested less often and cases may go unreported (World Health Organization (WHO) [40]), allowing the virus to reach more susceptible groups such as the elderly and immune-compromised. Recent reports have also suggested that the Omicron variant and its sub-lineages may lead to more frequent hospitalization in children, as children make up a larger part of patients hospitalized with COVID-19 than in previous infection waves caused by other variants of concern [41]. Moreover, when considering that SARS-CoV-2 RNA could be detected in a nursing home facility, it puts into question the prevention guidelines in place at the time, when it comes to the safety of the elderly population, as previous epidemiological data from Norway show that the majority of COVID-19 related deaths are among this risk group [48].

However, evidence to date still suggests that children are not among the main drivers of the pandemic [49]. That added to the fact that asymptomatic children have significantly lower viral loads compared to children with symptomatic infections [50] have resulted in less effort trying to understand the role of children in the airborne transmission of SARS-CoV-2 [51].

In another study, it was highlighted that although estimates of children’s susceptibility and infectivity are lower than those of adults within a household, it is important to remember that their role in the spread of SARS-CoV-2 is also affected by different contact patterns and hygiene habits outside the household (Dattner et al. [46]). More intense contact and mixing among children compared to adults in schools, e.g., could offset the effect of reduced susceptibility. In a review it is brought to attention the fact that roughly half of the United States population goes to school, works in a school, or is a first-degree contact of individuals that frequent these environments, suggesting that in-school transmission can impact the disease burden in surrounding communities [52]. This raises the alarm for the higher probability of virus spreading in school settings, as they have one of the main elements for a superspreading event, which is prolonged indoor exposure to other people. Whether these schools have appropriate ventilation or not is another factor that should be taken into consideration when evaluating exposure risk in school settings. Another important factor concerning children and increased risk of SARS-CoV-2 infection is that households with children in low income, urban communities have an extremely high household secondary attack rate, with children playing important roles as index cases [51].

This study sheds light on the presence of SARS-CoV-2 in indoor and outdoor environments, particularly focusing on healthcare and non-healthcare settings. With lifted COVID-19 restrictions and a significant percentage of the population vaccinated, our findings provide valuable insights into the dynamics of SARS-CoV-2 transmission at a time where all restrictions have been lifted and vaccination coverage is high.

Our air sampling in Norway took place in two cities, namely Oslo and Ås, in the end of April and beginning of May 2022. During this period, there had been a steady decline in the number of new patients admitted to Norwegian hospitals with COVID-19, with the number of new patients admitted per 100.000 people being highest in the age groups 75–84 and ≥ 85 years [19]. Of note, during this period, 58% of people who deceased due to COVID-19 complications died in a health institution other than a hospital, primarily in nursing homes. The Omicron variant BA.2 was the dominant sub-lineage in the country, accounting for 95–100% of all whole-genome sequenced samples during that period [19].

Considering that our sampling campaign took place at a moment of low-transmission in Norway, and that sampling covered various indoor and outdoor environments (public parks, public transport stations, university restaurants, a nursing home, a kindergarten, and a health clinic), the fact that SARS-CoV-2 RNA could be detected only in environments related to children and the elderly raises some issues when it comes to mitigation guidelines and prevention strategies.

First of all, as vaccination coverage is increasing around the world and we have started to normalize life towards the pre-COVID-19 time, COVID-19 cases are escalating and SARS-CoV-2 surveillance is reduced, painting out a coming challenging winter in Europe [53]. That being said, it is urgent that countries in the EU region relaunch mitigation efforts and are ready to respond to an increased burden on their health-care systems.

The application of what the WHO calls “Five Pandemic Stabilizers” (increased vaccination rates, a second booster dose to immunocompromised people and their close contacts, mask wearing indoors and in public transports, improving ventilation in crowded and public spaces, and applying rigorous therapeutic protocols for those at risk of severe disease) will be of the utmost importance in order to control virus transmission during autumn and winter [53].

When considering that children still represent the majority of the unvaccinated people not only in the EU region but in a global scale, and the fact that this group is often asymptomatic and less frequently tested, special attention should be given to the importance of re-implementing mask wearing indoors and in public transports, as this is still one of the most efficient interventions against SARS-CoV-2 airborne transmission [54, 55]. This would reduce transmission of the virus to children, which in turn would help prevent the virus spread from this group to at-risk groups such as the elderly and immunocompromised. Furthermore, considering the surge of Omicron and its sub-lineages that are more easily transmissible, reinstating mask mandates might be the best strategy to control community transmission of COVID-19 [55].

## Conclusion

This study detected SARS-CoV-2 RNA in air samples from specific locations, such as a kindergarten, a nursing home, and a pediatrics waiting room in a health clinic. Importantly, the RNAse pre-treatment indicated that the virus in these samples was likely non-infectious, suggesting that the collection process may have contributed to the inactivation of the virus. Despite this, genetic sequencing revealed mutations associated with increased infectivity and virulence, raising concerns about the potential risks associated with infection. One notable aspect highlighted by this study is the role of children in the transmission of SARS-CoV-2, particularly in settings with prolonged indoor exposure. Children, even if they experience milder symptoms, can still play a crucial role in spreading the virus to more vulnerable populations, such as the elderly. This emphasizes the importance of continued vigilance and public health measures, especially given the uncertainty surrounding new variants like Omicron and its sub-lineages. As we move forward, the study underscores the need for a balanced approach to COVID-19 prevention and control. Vaccination efforts are crucial, but it’s essential to recognize that children, especially the younger ones, often remain unvaccinated and can still contribute to virus transmission. Therefore, reinstating mask mandates, improving ventilation in public spaces, and implementing rigorous therapeutic protocols are recommended as part of a comprehensive strategy to control the virus, particularly during the autumn and winter months. This research serves as a timely reminder that the fight against COVID-19 is ongoing, and we must adapt our strategies to evolving circumstances, including emerging variants and the role of different demographic groups in transmission. It underscores the importance of maintaining a multifaceted approach to minimize the risks associated with the virus and protect public health.

## Data Availability

The data presented in this study are available on request from the corresponding author.
